# Passive exposure to e-cigarette emissions is associated with worsened mental health

**DOI:** 10.1186/s12889-022-13470-9

**Published:** 2022-06-07

**Authors:** Kayla Rae Farrell, Michael Weitzman, Emma Karey, Teresa K. Y. Lai, Terry Gordon, Shu Xu

**Affiliations:** 1 Department of Environmental Medicine, New York University Grossman School of Medicine, New York, NY 10010 USA; 2 Department of Social and Behavioral Sciences, New York University School of Global Public Health, New York, NY 10003 USA; 3 Department of Pediatrics, New York University Grossman School of Medicine, New York, NY 10016 USA; 4 Department of Biostatistics, New York University School of Global Public Health, 708 Broadway, 7th floor - Rm 761, New York, NY 10003 USA

**Keywords:** E-cigarette use, Smoking, E-cigarette emissions, Secondhand smoke, Internalizing disorders, Mental health

## Abstract

**Background:**

Cigarette smoking, secondhand cigarette smoke (SHS) exposure, and e-cigarette use (“vaping”) are each associated with increased rates of depressive symptoms and other internalizing mental health disorders. The prevalence of vaping has increased greatly, yet the mental health correlates of secondhand exposure to e-cigarette emissions are as yet to be investigated. This study examined the potential adverse mental health outcomes associated with different tobacco exposures (direct and passive), with a particular focus on the mental health correlates of secondhand exposure to e-cigarette emissions.

**Methods:**

The Population Assessment of Tobacco and Health Study data collected from a sample of 16,173 Wave 4 adults were used to test the hypothesis that secondhand e-cigarette emissions exposure is associated with increased odds of internalizing mental health disorders. Individuals were categorized as exclusive cigarette smokers, exclusive e-cigarette users, cigarette and e-cigarette dual users, exclusive noncombustible tobacco users, secondhand smoke exposed non-users, secondhand e-cigarette emissions exposed non-users, and non-users with no current SHS/secondhand e-cigarette aerosol exposure. Adjusted weighted logistic regression analysis was used to investigate the association between exposure type and internalizing problems as assessed by scores on the Global Appraisal of Individual Needs-Short Screener (GAIN-SS), a widely used instrument for assessing mental health problems.

**Results:**

Cigarette smokers (AOR = 2.53, 95% CI: 2.19–2.92), e-cigarette users (AOR = 3.14, 2.41–4.09), dual users (AOR = 3.37, 2.85–4.00), noncombustible tobacco users (AOR = 1.48, 1.01–2.17), SHS exposed non-users (AOR = 1.63, 1.37–1.94), and secondhand e-cigarette emissions exposed non-users (AOR = 1.43, 1.03–1.99) were each associated with increased odds of moderate to severe internalizing mental health problems as compared to unexposed non-users. Odds of internalizing problems among SHS and secondhand e-cigarette emissions exposed non-users did not differ (*p* = 0.46).

**Conclusions:**

This is the first study, to our knowledge, to identify an association between recent secondhand exposure to e-cigarette emissions and mental health problems, and the risk is comparable to that of SHS. Corroboration of this relationship needs further research to explicate directionality and mechanisms underlying this association.

**Supplementary Information:**

The online version contains supplementary material available at 10.1186/s12889-022-13470-9.

## Background

Tobacco use, principally as cigarette smoking, continues to be the leading preventable cause of premature death globally [1]. While cigarette use in the US continues to decline, there has been a remarkably rapid uptake of electronic cigarette (e-cigarette) use, called “vaping” [2], since their introduction to the US market in 2007 [3]. Despite a decline in overall e-cigarette use in 2020, it remains exceedingly popular among tobacco-naive middle and high school aged adolescents, and the Centers for Disease Control and Prevention notes that “youth e-cigarette use remains an epidemic” [4]. The widespread adoption of vaping by adolescents has led to a great concern that e-cigarettes may foster a new wave of nicotine dependence, thereby endangering the successes of more than a half century of tobacco control efforts [3].

The risks associated with e-cigarette use remains controversial within the scientific and public health communities [5]. Debate has largely centered on their potential to aid in harm reduction (i.e., whether they are a safer alternative to cigarette smoking and whether their use will abate cigarette use) [6, 7], as well as their potential to enlist tobacco-naïve adolescents and young adults to become dependent on nicotine via new nicotine delivery devices [8]. In addition, there remains a paucity of evidence regarding the potential for e-cigarette use in causing adverse acute and long-term physical and mental health consequences. Even less currently is known about the potential adverse health effects of exposure to secondhand e-cigarette emissions. This is particularly troubling given the wide ranging and pervasive nature of adverse effects associated with secondhand smoke (SHS) exposure and the prevailing perception by the general public that passive exposure to e-cigarette emissions is safe—a premise that has been reinforced by e-cigarette advertising campaigns and media coverage [9–11].

Mental health problems, well recognized to be among the most pervasive and pernicious of public health problems, can dramatically impair quality of life and overall health. “Internalizing disorders” is a term commonly used by mental health professionals—and referenced in the Diagnostic and Statistical Manual of Mental Disorders-5 (DSM-5)—to denote a major category of emotional and behavioral problems characterized by symptoms and feelings of depression, anxiety, and/or social withdrawal [12, 13]. Externalizing problems, in contrast, are characterized by aggressive, oppositional defiant and anti-social emotional states and behaviors [13]. Internalizing problems present a significant disease burden worldwide: depressive disorders and anxiety disorders, which are highly interrelated and often co-occur [14], are very common psychiatric problems [15]. It is estimated that 20% of the US population will suffer a depressive disorder at some point during their lifetime [14]. In 2019, the global burden of depressive disorders impacted 279 million people, leading to 46.8 million Disability-Adjusted Life Years (DALYs) [16]; similarly, anxiety disorders affected 301 million people worldwide, leading to 28.7 million DALYs [16].

It is well established that cigarette smokers are at increased risk for mental health disorders [17–19]. Similarly, those exposed to SHS have increased depressive and other internalizing problems. It has been shown that non-smokers reporting substantial SHS exposure are nearly 50% more likely to experience psychological distress [20]. The relationship between SHS exposure and increased rates of internalizing problems such as depression, anxiety, and panic attacks has been found in the US [21, 22], Korea [23–28], Canada [29], Germany [30], Japan [31, 32], and China [33, 34], with some demonstrating dose-response relationships [34]. A growing literature suggests that vaping e-cigarettes is independently associated with increased rates of depressive symptoms specifically [35–39], and internalizing disorders broadly [40–45]. To our knowledge, no research has: 1) examined the relationship between internalizing mental health disorders among persons with no history of tobacco product use but who are passively exposed to e-cigarette emissions, or 2) directly compared the odds of internalizing problems among consumers of different tobacco products. Improved understanding of the association between different tobacco exposures (via consumption or passive inhalation) and potential adverse mental health outcomes may have substantial implications both for mental health interventions and for tobacco control efforts.

The aim of the current study is to use a nationally representative dataset to investigate potential adverse mental health outcomes associated with different tobacco exposures (direct and passive), with a particular focus on secondhand exposure to e-cigarette emissions. We hypothesized that passive exposure to e-cigarette emissions, as well as e-cigarette consumption (i.e., vaping) is associated with an increased risk of internalizing mental health disorders.

## Methods

The Population Assessment of Tobacco and Health (PATH) Study is a nationally representative, cohort study of tobacco products and associated health outcomes. Secondary analyses of adult data (≥ 18 years old) from Wave 4 (December 2016–January 2018) of the PATH study (*n* = 16,173) were conducted. The fourth wave was used as it was the most recently available data for public use at the time of analysis. Details regarding the PATH study design are available online [46].

### Tobacco use/exposure status

Two types of tobacco product exposure were evaluated: that resulting from direct tobacco product consumption, and that resulting from passive exposure to tobacco product emissions. Tobacco product status was determined based on use or exposure at least once within the past 30 days.

Subjects were assigned to a binary tobacco use/exposure category based on responses to queries about former and current tobacco product use or passive exposure. Former smoking status was determined using the PATH derived variable indicating former established cigarette smokers who have smoked at least 100 cigarettes in their lifetime, but had not smoked within the past 12 months. Current tobacco product use was determined based on responses to questions regarding cigarette smoking (“In the past 30 days, have you smoked a cigarette, even one or two puffs?”), e-cigarette use (“In the past 30 days, have you used an electronic nicotine product, even one or two times?”), and noncombustible tobacco use (“Have used smokeless tobacco/snus pouches/dissolvable tobacco within the past 30 days”). The exclusive e-cigarette use and non-combustible tobacco use groups were further categorized by former smoking status, as previously defined, to differentiate between those with and without a history of smoking.

SHS exposure (either residential or occupational) was determined based on self-reports of recent exposure (“Today”, “In the past week,” “In the past two weeks,” or “In the past month”) to the question “How recently did someone smoke around you while you were at work?” or the selection of a combustible tobacco product (‘smoke cigarettes, cigars, cigarillos or filtered cigars and pipe tobacco’) in response to the question: “Does anyone who lives with you now do any of the following…?”). Passive residential or occupational e-cigarette exposure was determined based on self-reports of recent exposure (“Today”, “In the past week,” “In the past 2 weeks,” or “In the past month”) to the question “How recently did someone use e-cigarettes or other electronic nicotine products around you while you were at work?” or the selection of exclusive e-product use when asked: “Does anyone who lives with you now do any of the following…?”

Non-users were defined as individuals who reported no current tobacco product use, no exposure to tobacco product emissions (residential or occupational), and no history of cigarette smoking.

**Fig. 1:**
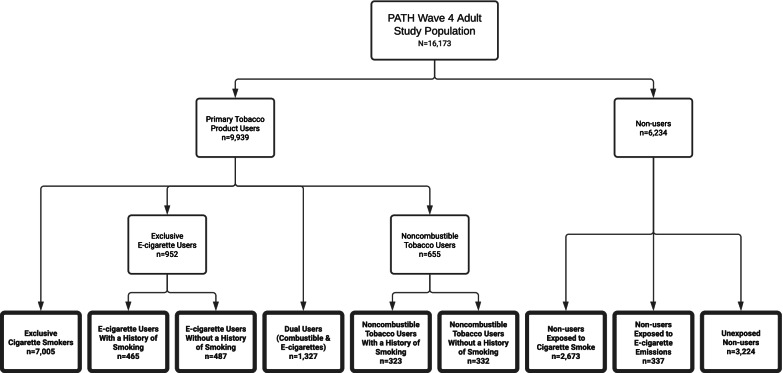
Categories of Tobacco Use/Exposure Status^†^ in the Wave 4 Adult Sample of the PATH Study Note: ^†^Bolded boxes indicate the nine groups actually included in the analyses

Therefore, nine categories were identified (Fig. 1): (1) Exclusive cigarette smoker (*n* = 7005), (2) exclusive e-cigarette user with no history of smoking (*n* = 487), (3) exclusive e-cigarette user with a history of smoking (*n* = 465), (4) dual user of combustible cigarettes and e-cigarettes (*n* = 1327), (5) exclusive noncombustible tobacco user with no history of smoking (*n* = 332), (6) exclusive noncombustible tobacco user with a history of smoking (*n* = 323), (7) non-user exposed to passive SHS (*n* = 2673), (8) non-user exposed to passive e-cigarette emissions (*n* = 337), and (9) non-user with no passive exposure to tobacco product emissions (*n* = 3224).

### Internalizing mental health disorders

Internalizing mental health problems were assessed using the Global Appraisal of Individual Needs-Short Screener (GAIN-SS), a diagnostic tool that screens for internalizing and externalizing problems that has been validated for clinical decision-making cut-points with great sensitivity and for disorder identification with great specificity [47, 48]. The GAIN-SS probes four items associated with internalizing disorders: 1) feeling very trapped, lonely, sad, blue, depressed, or hopeless about the future; 2) sleep trouble such as bad dreams, sleeping restlessly, or falling asleep during the day; 3) feeling very anxious, nervous, tense, scared, panicked, or like something bad was going to happen; and 4) becoming very distressed and upset when something reminded you of the past [49]. The number of items endorsed over the past 12 months are summed to yield an internalizing problem score between 0 and 4, which is used to determine severity: low (0–1 symptom), moderate (2–3 symptoms), or high (4 symptoms) [47, 49–52]. Individuals with a score = 4 were characterized as having severe internalizing problems. For the purposes of our analyses, individuals with an internalizing GAIN-SS score ≥ 2 were characterized as having moderate to severe internalizing problems.

### Covariates

Participants reported socio-demographic characteristics including sex (male / female), age group (18–34 years / 35–55 years / ≥55 years), race/ethnicity (non-Hispanic white / non-Hispanic black / Hispanic / non-Hispanic other), and annual household income (<$49,999 / $50,000 - $99,999/ above $100,000). Body mass index (BMI) was derived via questionnaire data and categorized as underweight (below 18.5), normal or healthy weight (18.5–24.9), overweight (25.0–29.9), and obese (30.0 and above) [53]. Chronic condition was categorized by self-report of a professional diagnosis for any chronic conditions (i.e. heart disease, diabetes, cancer, chronic obstructive pulmonary disease, and stroke) in the past 12 months [54]. We excluded 59 participants with missing data on covariates.

### Statistical analyses

Univariate data analyses were conducted to examine the frequency and proportion of study variables. Bivariate data analyses were performed to assess the association between internalizing problems and potential covariates. Logistic regression analyses were conducted to assess the association between tobacco product use status/secondhand exposure type and moderate to severe internalizing problems (GAIN-SS scores ≥2), adjusted for covariates. To study how sensitive the association between tobacco product use/exposure type and internalizing problems was to the cut-point of GAIN-SS score, we conducted two additional logistic regression analyses using the cut-points ≥ 3 and = 4. Adjusted odds ratios (AORs) and the 95% confidence intervals (95% CI; estimated using balanced repeated replication method) are reported [55]. Sampling weights were implemented in all analyses unless indicated otherwise. Weighting procedures were used to correct for differential probability of survey selection, nonresponse, and sampling frame bias; therefore, the weighted sample represents the US adult population at the time of data collection. All data analyses were performed using SAS software version 9.4 (SAS Institute Inc., Cary, NC, USA).

## Results

### Univariate and bivariate analyses

Descriptive statistics for characteristics of the study sample and the results of bivariate analyses are presented in Table 1. Most participants were younger than 55 years of age (77.1%) and were non-Hispanic White (62.4%). Approximately half of the sample were women (49.9%). One third of participants scored moderate-to-severe (≥2) on the GAIN-SS (34.8%). Fewer than half of these individuals had a GAIN-SS = 4 (15.4%). Moderate to severe internalizing GAIN-SS scores were associated with each category of tobacco use and exposure status, age, race/ethnicity, sex, annual family income, BMI, and chronic disease conditions in the past 12 months. Crude odds ratios for moderate to severe internalizing problems are presented in Table S1.

**Table 1:** Characteristics of the PATH Wave 4 Adult Sample (*N* = 16,173) and Bivariate Association^a^ Between Moderate to Severe Internalizing Problems and Study Variables

Characteristic	Total Sample	GAIN-SS < 2	GAIN-SS ≥ 2
	N (weighted %)	N (weighted %)	N (weighted %)
Age			
18–34 years	8626 (37.4%)	4462 (56.6%)	4164 (43.4%)
34–54 years	4675 (39.7%)	2777 (68.5%)	1898 (31.5%)
≥55 years	2872 (22.9%)	1951 (73.5%)	921 (26.5%)
Sex			
Male	7666 (50.1%)	4857 (70.4%)	2809 (29.6%)
Female	8507 (49.9%)	4333 (60.0%)	4174 (40.0%)
Race/Ethnicity			
Non-Hispanic White	9583 (62.4%)	5272 (63.3%)	4311 (36.7%)
Non-Hispanic Black	2270 (12.4%)	1426 (67.8%)	844 (32.2%)
Hispanic	3041 (16.2%)	1792 (67.4%)	1249 (32.6%)
Non-Hispanic Other	1279 (9.0%)	700 (70.6%)	579 (29.4%)
Annual Income Level			
<$49,999	9615 (50.4%)	5081 (58.7%)	4534 (41.3%)
$50,000 - 99,999	3422 (25.1%)	2112 (71.0%)	1310 (29.0%)
≥$100,000	2208 (18.6%)	1427 (72.4%)	781 (27.6%)
Missing Values	928 (5.9%)	570 (73.0%)	358 (27.0%)
Body Mass Index			
Underweight (< 18.5)	754 (4.4%)	395 (62.3%)	359 (37.7%)
Normal weight (18.5–24.9)	5769 (32.1%)	3236 (64.6%)	2533 (35.4%)
Overweight (25.0–29.9)	4789 (31.9%)	2912 (68.1%)	1877 (31.9%)
Obese (30+)	4861 (31.6%)	2647 (63.2%)	2214 (36.8%)
Chronic Diseases ^b^			
Yes	1721 (11.9%)	850 (57.5%)	871 (42.5%)
No	10684 (71.6%)	6279 (67.0%)	4450 (33.0%)
Missing Values	3768 (16.5%)	2061 (62.7%)	1707 (37.3%)
Tobacco Use/Exposure Group			
*Consumption Groups*			
Exclusive Noncombustible Tobacco User (with no smoking history)	332 (1.8%)	228 (72.7%)	104 (27.3%)
Exclusive Noncombustible Tobacco User (with smoking history)	323 (1.8%)	225 (72.3%)	98 (27.7%)
Exclusive Cigarette Users	7005 (33.2%)	3798 (57.1%)	3207 (42.9%)
Exclusive E-cigarette Users (with no smoking history)	487 (1.5%)	214 (43.7%)	273 (56.3%)
Exclusive E-cigarette Users (with smoking history)	465 (2.3%)	267 (59.7%)	198 (40.3%)
Dual Users	1327 (5.5%)	568 (45.7%)	759 (54.3%)
*Exposure Groups*			
Secondhand Smoke Exposed Non-users	2673 (20.4%)	1508 (66.0%)	1165 (34.0%)
Secondhand E-cigarette Aerosol Exposed Non-users	337 (2.4%)	205 (68.9%)	132 (31.1%)
*Reference Group*			
Unexposed Non-users	3224 (31.1%)	2177 (77.0%)	1047 (23.0%)
Internalizing Problems: GAIN-SS Score at Various Cut-points			
≥2	6983 (34.8%)	–	–
≥3	5153 (24.9%)	–	–
4	3343 (15.4%)	–	–

^a^ Bivariate analyses involved Person’s Chi-squared test for unweighted data and Rao-Scott Chi-squared test for weighted data. Results indicated that moderate to severe internalizing GAIN-SS scores were associated with each category of tobacco use and exposure status, age, race/ethnicity, sex, annual family income, BMI, and chronic disease conditions in the past 12 months

^b^ Self-reported diagnosis of the following chronic conditions in the past 12 months: heart disease, diabetes, cancer, chronic obstructive pulmonary disease, and stroke. A subgroup of participants reported lifetime chronic conditions instead of diagnosis in the past 12 months. They were characterized as the “Missing Values” group so as to be kept in analysis

### Logistic regression analyses

**Fig. 2:**
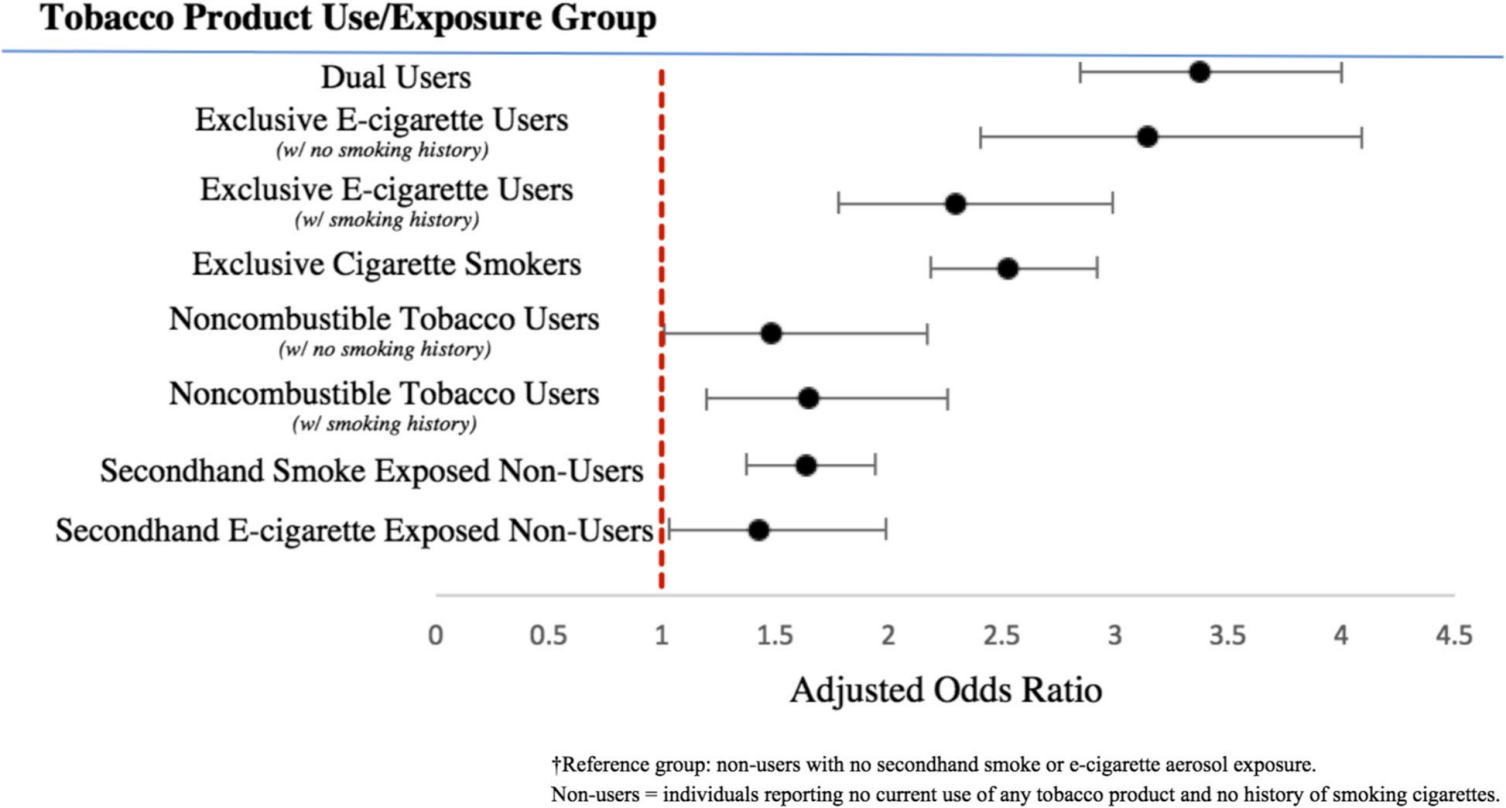
Forest Plot for Adjusted Odds Ratios with 95% Confidence Intervals of Moderate to Severe Internalizing Problems for Tobacco Product Use/Exposure Groups^†^ Note: All adjusted odds ratios are statistically significant (*p* < 0.05)

Weighted logistic regression models were conducted to examine the associations between tobacco product use or exposure status (nine categories in Fig. 1) and internalizing problems, adjusting for covariates (Fig. 2). Compared to non-users with no passive exposure to tobacco product emissions, dual tobacco product users (i.e., persons who use both cigarettes and e-cigarettes) had the highest odds of moderate to severe internalizing problems (AOR = 3.37, 95% CI: 2.85, 4.00). Exclusive e-cigarette users with no history of smoking were at greater risk for a GAIN-SS ≥2 than were exclusive cigarette smokers (AOR = 3.14; 95% CI: 2.41, 4.09 and AOR = 2.53; 95% CI: 2.19, 2.93, respectively), although these odds were not statistically different (*p* = 0.09). However, exclusive e-cigarette users that reported a smoking history were lower still (AOR = 2.30, 95% CI: 1.78, 2.99). Risk of moderate to severe internalizing problems (GAIN-SS ≥2) among noncombustible tobacco users were lower than that of individuals who currently use an inhalable tobacco product, regardless of whether they had a history of cigarette smoking (AOR = 1.65, 95% CI: 1.2, 2.26) or not (AOR = 1.48, 95% CI: 1.01, 2.17).

Among non-users, the odds of moderate to severe internalizing problems (GAIN-SS ≥2) were higher for those passively exposed to tobacco product emissions, regardless of the tobacco product source, as compared with non-users with no passive exposure to tobacco product emissions (SHS exposure AOR = 1.63, 95% CI: 1.37, 1.94; e-cigarette aerosol exposure AOR = 1.43, 95% CI: 1.03, 1.99) (Fig. 2).

Results also indicate that moderate to severe internalizing problems were associated with younger age, female sex, non-Hispanic White race/ethnicity, lower annual household income, and diagnosis of chronic disease in the past 12 months, but not BMI (see Table S1).

After assessing the additional cut-points for internalizing problems, we found consistent results that any category of tobacco product use or exposure is associated with an increased likelihood of internalizing problems (Table 2). At the two most severe cut-points for internalizing problems, however, the differences between non-users passively exposed to e-cigarette emissions and the non-user reference group do not reach statistical significance. Regardless of cut-point, dual use always had the highest AOR, and there was no difference in AORs between those exposed to secondhand smoke or e-cigarette emissions. In addition, the relative elevated risk for internalizing problems for each tobacco product use/exposure category followed a similar rank order.

**Table 2:** Adjusted Odds Ratios^a^ for Internalizing Problems for Different Tobacco Product Use/Exposure Status

	GAIN-SS ≥ 2	GAIN-SS ≥ 3	GAIN-SS = 4
	AOR (95% CI)	AOR (95% CI)	AOR (95% CI)
Non-users^b^ with no secondhand exposure	Ref	Ref	Ref
Exclusive Cigarette Smokers	2.53 (2.19, 2.92)*	2.40 (2.02, 2.85)*	2.72 (2.18, 3.39)*
Exclusive E-cigarette Users *(with no history of smoking)*	3.14 (2.41, 4.09)*	2.73 (2.13, 3.51)*	3.37 (2.44, 4.67)*
Exclusive E-cigarette users *(with history of smoking)*	2.30 (1.78, 2.99)*	2.21 (1.69, 2.90)*	2.48 (1.76, 3.48)*
Dual users	3.37 (2.85, 4.00)*	3.52 (2.91, 4.25)*	4.16 (3.23, 5.35)*
Noncombustible Tobacco Users *(with no history of smoking)*	1.48 (1.01, 2.17)*	1.38 (0.91, 2.10)	1.46 (0.84, 2.55)
Noncombustible Tobacco Users *(with a history of smoking)*	1.65 (1.2, 2.26)*	1.51 (1.02, 2.24)*	1.54 (0.87, 2.71)
Secondhand Smoke Exposed Non-users^b^	1.63 (1.37, 1.94)*	1.49 (1.21, 1.84)*	1.67 (1.29, 2.16)*
Secondhand E-cigarette Emissions Exposed Non-users^b^	1.43 (1.03, 1.99)*	1.21 (0.83, 1.76)	1.43 (0.86, 2.39)

*AOR* adjusted odds ratio, *CI* confidence interval. AORs marked with an asterisk (*) are statistically significant (*p* < 0.05)

^a^Adjusted for the following covariates: sex, age, race/ethnicity, chronic conditions, BMI, and annual household income

^b^Non-users = individuals reporting no current use of any tobacco product and no history of smoking cigarettes

## Discussion

This is the first study that we are aware of to identify an association between recent exposure to secondhand e-cigarette emissions and moderate to severe internalizing problems, and the risk was found to be comparable to that of SHS exposure in this large, nationally representative sample of the US adult population. Consistent with the literature on combustible cigarette smoking [56–60], SHS exposure [20–34], and more recently e-cigarette use [35, 39, 61, 62], the data in the current study indicate that every category of tobacco product use or exposure assessed was associated with an increased likelihood of mental health problems. Of all categories of tobacco use/exposure, dual users of combustible and e-cigarettes had the highest risk. These associations remained significant even after controlling for multiple other characteristics that are known to be highly associated with internalizing problems such as sex, race/ethnicity, age, chronic health conditions, annual income, and BMI [63–67].

Similar to the results presented here, earlier population-based cross-sectional studies have found that smoking is associated with internalizing disorders, such as depression [68] and anxiety [58–60, 69], and that the prevalence of cigarette smoking is higher among those with psychiatric diagnoses when compared to the general population [17–19]. Previous research has attempted to interpret such findings by proposing that users with emotional dysfunctions depend on cigarettes to self-medicate [70–72], as depressed individuals have been found to smoke at increased rates and with greater intensity due to low positive affect, high negative affect and cognitive impairment [56]. Currently, there is a lack of consensus on the directionality of this relationship, i.e. some longitudinal studies have found that tobacco use precedes depressive and anxiety disorders [73–77]; whereas others have found evidence that depression is associated with future tobacco use [78]. Still other studies have found evidence of a bidirectional relationship, where both tobacco use and internalizing mental health disorders are independent risk factors for one another [79, 80]. Well-designed, longitudinal research is needed to further investigate the critical issue of directionality, with a particular focus on e-cigarette use.

A more recent and limited literature has found that similar to smoking, e-cigarette use appears to be associated with increased levels of psychological distress, possibly in a dose-response fashion [81]. Increasing evidence is accumulating that illustrates a relationship between e-cigarette use and internalizing disorders [40–45], and more specifically e-cigarette use and depression among adults [35–39] and children/adolescents [82–85]. Whether e-cigarette use is associated with anxiety disorders is less well characterized [86, 87]. Dual use of combustible and electronic cigarettes has been consistently linked to depressive/internalizing disorders [36, 43, 61, 83, 88, 89]. Findings from the current study provide further evidence in support of an independent link between e-cigarette use and internalizing disorders, as well as evidence that dual users have the greatest risk. These data add to the growing body of literature that e-cigarette use may not be a harm-free alternative to smoking.

Another concern of great public health importance associated with cigarette smoking is the increased risk of mental health problems among those with SHS exposure [90]. SHS contains over 4000 chemicals, including toxic compounds like hydrogen cyanide, heavy metals such as lead and chromium, as well as a wide range of organic compounds [91], each entailing a unique profile and mechanism of potential health consequences. SHS exposure has been found to be associated with psychological distress [20], depressive conditions [24, 25, 27, 29–34, 92, 93], and panic attacks [22] in studies conducted in the US and around the world. The findings of the current study confirm those of these earlier studies.

No study to date that we are aware of has investigated the relationship between exposure to passive e-cigarette aerosols and adverse mental health outcomes. Akin to cigarette smoke, e-cigarette emissions have been found to include harmful substances like heavy metals (e.g. lead, chromium and nickel) [94–96], ultrafine particles [97], and inorganic [98] and volatile organic compounds [97]. Although the concentrations of chemical compounds in e-cigarette emissions have been found to be lower than concentrations of toxicants in SHS, some constituents of e-cigarette emissions are known carcinogens [99, 100]. Furthermore, some e-cigarette devices have even been found to emit metals and nicotine quantities that exceed those of combustible cigarettes [101, 102]. Despite the identification of such toxic constituents, the general public is not well informed of potential acute or long-term risks of secondhand exposure, and the information about untoward exposures and outcomes from secondhand e-cigarette exposure is still under active investigation. In the US, 40% of adults believe secondhand e-cigarette exposure causes “little harm” or only “some harm” and 33% are unsure of the potential dangers [11]. This can be compared to the fact that 64.5% of US adults perceive SHS as “very harmful,” and the fact that dual users report a preference for vaping rather than smoking in the presence of loved ones [103, 104]. Misconceptions about the risks associated with e-cigarette emissions put exposed non-users, including children, at risk. The fact that adult non-users passively exposed to e-cigarette emissions at home or in the workplace were almost 1.5 times more likely to have moderate to severe internalizing problems, warrants further study to determine if in fact this relationship is causal in nature, i.e. that exposure to these emissions actually causes or contributes to an increased burden of mental health problems. Of particular note, these data suggest that the risk of moderate to severe internalizing problems among those passively exposed to e-cigarette emissions is no different from that of those exposed to SHS.

Previous PATH studies demonstrated that internalizing and externalizing problems were associated with the use of cigarettes, e-cigarettes, or their dual use, in both adults and youth [49, 51, 105]. Some of these other studies used different cut-points for internalizing problems and focused on different groups of tobacco product use. We were particularly interested in moderate to severe internalizing problems (GAIN-SS ≥2) as opposed to just those with severe internalizing problems (GAIN-SS =4) based on clinical evidence that even those with moderate internalizing problems benefit from mental health intervention/treatments [47, 48, 50]. Furthermore, consistent with extensive earlier literature, the findings of this study corroborate the independent association of younger age [66], female sex [63], non-Hispanic White race/ethnicity [67], lower annual household income [64], and diagnosis of chronic disease in the past 12 months [106] with internalizing problems.

Limitations of this study should be noted. All analyses were cross-sectional; thus it is impossible to determine the directionality of the association between tobacco product use and exposure and internalizing problems. While it may seem improbable that internalizing disorders lead to secondhand tobacco product exposure, it is possible that people with poor mental health have a higher likelihood of working or living with other individuals who are tobacco product users, and as a result be more likely to experience secondhand emissions themselves. Moreover, non-smoking/non-vaping participants who live and/or work with smokers and/or e-cigarette users may feel a sense of isolation within their homes or the workplace that may ultimately contribute negatively to their mental health. Without knowing the length of time that subjects smoked, vaped, or experienced secondhand exposure; the intensity and frequency or the exact nature of the product used or exposed to; or the length or frequency with which subjects experienced symptoms of mental health problems, it is not possible to know how such differences might have influenced the observed associations. Very limited exposure (i.e., being exposed to a coworker vaping once over a 30 day period), likely did not impact internalizing problems. Moreover, it is possible nicotine specifically plays a critical role in the exacerbation of internalizing problems among those who use and are exposed to tobacco products. However, nicotine content can vary greatly by e-cigarette device type and this was not addressed in the present study [102, 107]. Also, the scale used to assess internalizing mental health problems, GAINS-SS, did not allow us to distinguish among the various internalizing disorders (e.g., depression, anxiety disorder, post-traumatic stress disorder, etc). Additionally, the PATH dataset lacks important information regarding social context, such as the mental health of family members, something known to be associated with an individual’s mental health [108–111]. Also, secondhand exposure is only defined in the context of occupational and residential exposure and not social settings, which likely contribute to SHS and passive e-cigarette aerosol exposure for many individuals. Furthermore, the utilization of self-reported data for the categorization of tobacco product use/exposure may have been influenced by recall bias. Lastly, this study does not address the mechanisms underlying passive exposure to e-cigarette emissions and increased rates of internalizing mental health problems.

## Conclusions

Identifying and understanding potential associations between secondhand e-cigarette emissions and adverse health effects is of vital importance to the health of the public. These findings point to one such potential category of major health difficulties, namely an increased risk of mental health problems. In the case of SHS, smoking ban policies significantly reduce major depression risk among those who have never been smokers [112]. The findings reported in this paper indicate the marked need for further investigation of the safety profile of e-cigarette emission exposure, and whether smoking ban policies should extend to cover e-cigarettes, particularly in environments where vulnerable populations, including children, pregnant women, and those with chronic health conditions are likely to be exposed.

## Supplementary Information

**Additional file 1: Supplementary Table 1.** Crude and Adjusted^†^ Odds Ratios for Moderate to Severe Internalizing Problems.

## Data Availability

The dataset supporting the conclusions of this article is available in the Population Assessment of Tobacco and Health (PATH) study public-use files (ICPSR 36498), 10.3886/ICPSR36231.v28.

## Abbreviations

SHS: Secondhand smoke
PATH: The Population Assessment of Tobacco and Health Study
GAIN-SS: Global Appraisal of Individual Needs-Short Screener
AORs: Adjusted odds ratios
CI: Confidence intervals
BMI: Body mass index
