# Supplementary material for: Passive exposure to e-cigarette emissions is associated with worsened mental health
Source: BMC Public Health. 2022 Jun 7;22:1138. doi: 10.1186/s12889-022-13470-9 (PMC9172130; doi:10.1186/s12889-022-13470-9)
Supplement: Supplementary file 1 — Additional file 1: Supplementary Table 1. Crude and Adjusted† Odds Ratios for Moderate to Severe Internalizing Problems. [file 12889_2022_13470_MOESM1_ESM.docx]

**Supplementary Table 1:** Crude and Adjusted^†^ Odds Ratios for Moderate to Severe Internalizing Problems

|  | **GAIN-SS ≥2** | |
| --- | --- | --- |
| **Characteristic** | Crude OR (95% CI) | AOR (95% CI) |
| Tobacco Product Use/Exposure Groups |  |  |
| Non-users with no secondhand exposure | Ref | Ref |
| Exclusive Cigarette Smokers | 2.52 (2.23, 2.85)* | 2.53 (2.19, 2.92)* |
| Exclusive E-cigarette Users *(with no history of smoking)* | 4.32 (3.36, 5.57)* | 3.14 (2.41, 4.09)* |
| Exclusive e-cigarette users *(with history of smoking)* | 2.26 (1.78, 2.87)* | 2.30 (1.78, 2.99)* |
| Dual users | 3.99 (3.39, 4.69)* | 3.37 (2.85, 4.00)* |
| Noncombustible Tobacco Users *(with no history of smoking)* | 1.26 (0.89, 1.78) | 1.48 (1.01, 2.17)* |
| Noncombustible tobacco users *(with a history of smoking)* | 1.29 (0.95, 1.74) | 1.65 (1.2, 2.26)* |
| Secondhand Smoke Exposed Non-users | 1.73 (1.48, 2.03)* | 1.63 (1.37, 1.94)* |
| Secondhand E-cigarette Emissions Exposed Non-users | 1.52 (1.06, 2.18)* | 1.43 (1.03, 1.99)* |
| Age |  |  |
| 18-34 yrs | Ref | Ref |
| 34-54 yrs | 0.60 (0.53, 0.67)* | 0.56 (0.50, 0.64)* |
| ≥55 years | 0.47 (0.41, 0.54)* | 0.35 (0.30, 0.42)* |
| Sex |  |  |
| Male | 0.63 (0.58, 0.69)* | 0.59 (0.54, 0.66)* |
| Female | Ref | Ref |
| Race/Ethnicity |  |  |
| Non-Hispanic White | Ref | Ref |
| Non-Hispanic Black | 0.82 (0.73, 0.92)* | 0.72 (0.64, 0.82)* |
| Hispanic | 0.83 (0.75, 0.93)* | 0.73 (0.64, 0.83)* |
| Non-Hispanic Other | 0.72 (0.58, 0.89)* | 0.76 (0.61, 0.96)* |
| Annual Income Level |  |  |
| <$49,999 | 1.72 (1.53, 1.93)* | 1.44 (1.28, 1.64)* |
| $50,000 - 99,999 | Ref | Ref |
| ≥$100,000 | 0.93 (0.77, 1.12) | 1.11 (0.92, 1.35) |
| Missing Values | 0.91 (0.69, 1.20) | 0.89 (0.68, 1.18) |
| Body Mass Index |  |  |
| Underweight (<18.5) | 1.10 (0.87, 1.40) | 1.03 (0.80, 1.33) |
| Normal weight (18.5 - 24.9) | Ref | Ref |
| Overweight (25.0 - 29.9) | 0.85 (0.75, 0.97)* | 0.98 (0.86, 1.13) |
| Obese (30+) | 1.06 (0.94, 1.20) | 1.11 (0.98, 1.27) |
| Chronic Diseases |  |  |
| Yes | 1.51 (1.30, 1.74)* | 1.76 (1.48, 2.11)* |
| No | Ref | Ref |
| Missing Values | 1.21 (1.06, 1.39)* | 1.18 (1.03, 1.37)* |

Note: AOR: adjusted odds ratio; CI: confidence interval.

^†^ Adjusted for the following covariates: sex, age, race/ethnicity, chronic conditions, BMI, and annual household income

* p < 0.05
